# Incident Parkinson’s disease in kidney transplantation recipients: a nationwide population-based cohort study in Korea

**DOI:** 10.1038/s41598-021-90130-9

**Published:** 2021-05-18

**Authors:** Seon Ha Baek, Sehoon Park, Mi-yeon Yu, Ji Eun Kim, Sang Hyun Park, Kyungdo Han, Yong Chul Kim, Dong Ki Kim, Kwon Wook Joo, Yon Su Kim, Hajeong Lee

**Affiliations:** 1 Department of Internal Medicine, Hallym University Dongtan Sacred Heart Hospital, Hwaseong, Gyeonggi-do Republic of Korea; 2 Department of Biomedical Medicine, Seoul National University Hospital, Seoul, Republic of Korea; 3 Department of Internal Medicine, Armed Forces Capital Hospital, Seongnam, Gyeonggio-do Republic of Korea; 4 Department of Internal Medicine, Hanyang University Guri Hospital, Guri, Gyeonggio-do Republic of Korea; 5 Department of Internal Medicine, Korea University Guro Hospital, Seoul, Republic of Korea; 6 Department of Medical Statistics, College of Medicine, Catholic University of Korea, Seoul, Republic of Korea; 7 Department of Statistics and Actuarial Science, Soongsil University, Seoul, Republic of Korea; 8 Department of Internal Medicine, Seoul National University Hospital, 101 Daehak-ro, Jongno-gu, Seoul, 03080 Korea; 9 Kidney Research Institute, Seoul National University, Seoul, Republic of Korea

**Keywords:** Nephrology, Neurology

## Abstract

This nation-wide population based retrospective cohort study evaluated risk of incident Parkinson’ disease in kidney transplant (KT) recipients in Korea. From Korean National Health Insurance Service database, we identified incident KT recipients aged ≥ 40 years without any history of Parkinson’s disease between 2007 and 2015. We established two control cohorts without a history of Parkinson’ disease: (1) General population (GP) cohort of insured subjects without a history of kidney disease, (2) end-stage renal disease (ESRD) cohort of incident ESRD subjects, with frequency matched for age, sex, and inclusion year. Parkinson’s disease data were obtained from baseline until December 2017. We followed 8372 KT recipients, ESRD patients, and GP for 45,723, 38,357, and 47,476 patient-years, respectively. Their mean age was 51.2 years and 60.1% were men. During follow-up period, 19 KT recipients, 53 ESRD patients, and 15 GP developed Parkinson’ disease. Risk of incident Parkinson’s disease in KT recipients was similar to that in GP (adjusted hazard ratio [HR] 0.86, 95% confidence interval [CI] 0.35 to 2.13, *P* = 0.75) and significantly lower than that in ESRD patients (adjusted HR 0.31, 95% CI 0.18 to 0.52, *P* < 0.001). Older age was the strongest predictor for incident Parkinson’s disease in KT recipients.

## Introduction

Parkinson’s disease is the second most prevalent neurodegenerative disorder after Alzheimer’s disease and is characterized by bradykinesia, resting tremor, rigidity, and postural instability^1^. It has an estimated prevalence of 0.5% in the entire population and 1%-2% in individuals aged > 60 years^2^. Patients with Parkinson’s disease present with poor outcomes and it is associated with an increased risk of mortality compared with that in the healthy population (hazard ratio [HR] 1.5–2.7 times higher)^1^. The pathogenesis of Parkinson’s disease is thought to involve progressive neuron loss in brain structures, including the substantia nigra^3^. Previous epidemiological studies have reported several genetic and environmental risk factors for Parkinson’s disease^1,4,5^.

Several recent cohort studies have reported an association of chronic kidney disease (CKD)^6,7^ and end-stage renal disease (ESRD)^8^ with incident Parkinson’s disease. This is speculated to involve metabolic derangements, including hypoxia, uremic toxins, and metabolic acidosis with resulting persistent vasogenic edema in the basal ganglia and eventual permanent Parkinsonism characterized by cytotoxic derangements in the globus pallidus lesion^9–11^. However, a recent epidemiology study reported that patients undergoing tissue transplants, including kidney, heart, lung, and bone marrow transplantation, were at a lower risk of Parkinson’s disease than the general population^12^. They speculated that immunosuppressants may reduce the risk of Parkinson’s disease given the possible involvement of inflammation in the pathophysiology of Parkinson’s disease.

Kidney transplantation (KT) is considered the best treatment option for patients with ESRD mainly due to the resulting survival benefits and quality of life even with increase in age and comorbidities of contemporary transplant recipients^13–15^. Moreover, KT is becoming increasingly popular among older adults with ESRD with improvements in survival and graft loss in this population^16^. Given that KT recipients live longer with a functioning graft compared to dialysis-dependent patients with ESRD, they are at risk of incident age-related conditions, including neurodegenerative diseases. Therefore, there is a need to assess the effect of KT on the risk of incident Parkinson’s disease. In this study, we aimed to estimate the risk of incident Parkinson’s disease among KT recipients compared with patients with ESRD and the general population.

## Results

### Study population

**Figure 1:**
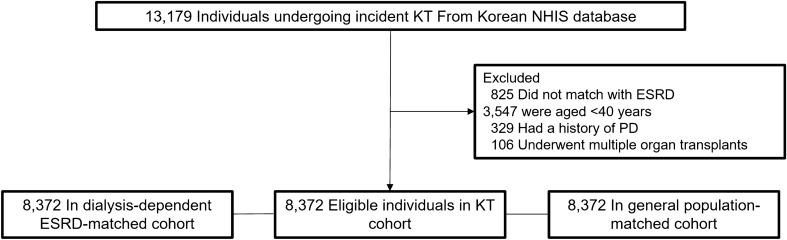
Study population. KT, kidney transplantation; NHIS, National Health Insurance Service; ESRD, end-stage renal disease; PD, Parkinson’s disease.

We followed 8372 KT recipients, dialysis-dependent patients with ESRD, and individuals in the general population for 45,723, 38,357, and 47,476 patient-years, respectively until the outcome date, death, or December 31, 2017 (Fig. 1). We performed among-group comparisons of the patients’ baseline characteristics (Table 1). Their mean age was 51.2 ± 7.0 years and men were 60.1%. There were lower and higher proportions of the KT recipients with Medical Aid coverage compared with dialysis-dependent patients with ESRD and the general population, respectively. The KT recipients had a similar and higher Charlson’s comorbidity index compared with dialysis-dependent patients with ESRD and the general population, respectively. KT recipients received preemptive KT in 29.8% and underwent hemodialysis mainly before KT. Further, 16.2%, 86.6%, and 82% of the KT recipients underwent pre-KT desensitization therapy, induction treatment using basiliximab, and immunosuppression maintenance using tacrolimus, respectively. KT recipients had a lower frequency of use of nonsteroidal anti-inflammatory drugs compared to that of dialysis-dependent patients with ESRD and the general population.

**Table 1:** Baseline characteristics of patients among the kidney transplants, dialysis-dependent ESRD, and general population groups.

Variables	Kidney transplant	Dialysis	General population	*P* value
	N = 8372	N = 8372	N = 8372	
Age, mean (SD), year	51.2 (7.0)	51.2 (7.0)	51.2 (7.0)	1
Male (%)	5028 (60.1)	5028 (60.1)	5028 (60.1)	
**Year grading, year (%)**				1
2007–2009	1802 (21.5)	1802 (21.5)	1802 (21.5)	
2010–2012	2914 (34.8)	2914 (34.8)	2914 (34.8)	
2013–2015	3656 (43.7)	3656 (43.7)	3656 (43.7)	
**Income (Q) (%)**				< 0.001
Medical Aid	1149 (13.7)	1936 (23.1)	253 (3.0)	
Q1	1587 (19.0)	2193 (26.2)	2186 (26.1)	
Q2	1492 (17.8)	1708 (20.4)	1833 (21.9)	
Q3	1720 (20.5)	1450 (17.3)	1870 (22.4)	
Q4	2424 (29.0)	1085 (13.0)	2230 (26.6)	
**CCI score (%)**				< 0.001
0	1 (0.01)	16 (0.2)	4652 (55.6)	
1–2	699 (8.3)	841 (10.0)	2839 (33.9)	
3–4	2552 (30.5)	2359 (28.2)	651 (7.8)	
≥ 5	5120 (61.2)	5156 (61.6)	230 (2.7)	
Diabetes mellitus (%)	4147 (49.5)	4147 (49.5)	718 (8.58)	< 0.001
Hypertension (%)	7699 (92.0)	7699 (92.0)	1867 (22.3)	< 0.001
Dyslipidemia (%)	4970 (59.3)	3791 (45.3)	1318 (15.7)	< 0.001
**Underlying disease (%)**				
Goodpasture’s syndrome	3 (0.04)	7 (0.08)	0 (0)	N/A
Microscopic polyangiitis	3 (0.04)	4 (0.05)	0 (0)	N/A
Systemic lupus erythematosus	118 (1.4)	85 (1.0)	0 (0)	N/A
REM sleep behavior disorder	98 (1.2)	86 (1.0)	0 (0)	N/A
**Previous dialysis modality (%)**				N/A
Preemptive	2498 (29.8)	0 (0)	8372 (100)	
Hemodialysis	3888 (46.5)	6350 (75.9)	0 (0)	
Peritoneal dialysis	1425 (17.0)	1556 (18.5)	0 (0)	
Mixed	561 (6.7)	466 (5.6)	0 (0)	
**Dialysis vintage period (%)**				N/A
< 5 y	6280 (75.0)	6111 (73.0)	8372 (100)	
≥ 5 y	2092 (25.0)	2261 (27.0)	0 (0)	
**Immunosuppressive treatment before inclusion (%)**				N/A
	1442 (17.2)	843 (10.1)	0 (0)	
**Immunosuppressive treatment after inclusion (%)**				N/A
Desensitization	1358 (16.2)	0 (0)	0 (0)	
**Induction**				
No use	348 (4.2)	8372 (100)	8372 (100)	
ATG	775(9.2)	0(0)	0(0)	
Basiliximab	7249(86.6)	0(0)	0(0)	
**Maintenance CNI**				
No use	201 (2.4)	8372 (100)	8372 (100)	
Tacrolimus	6867 (82.0)	0 (0)	0 (0)	
Cyclosporine	1304 (15.6)	0 (0)	0 (0)	
Use of NSAID (%)	1541 (16.4)	2116 (25.3)	2115 (25.3)	< 0.001

Abbreviation: CCI, Charlson’s comorbidity index; REM, rapid eye movement; ATG, anti-thymocyte globulin; CNI, calcineurin inhibitor; NSAID, nonsteroidal anti-inflammatory drugs.

### Between-group comparison of the incidence probability of Parkinson’s disease

**Figure 2:**
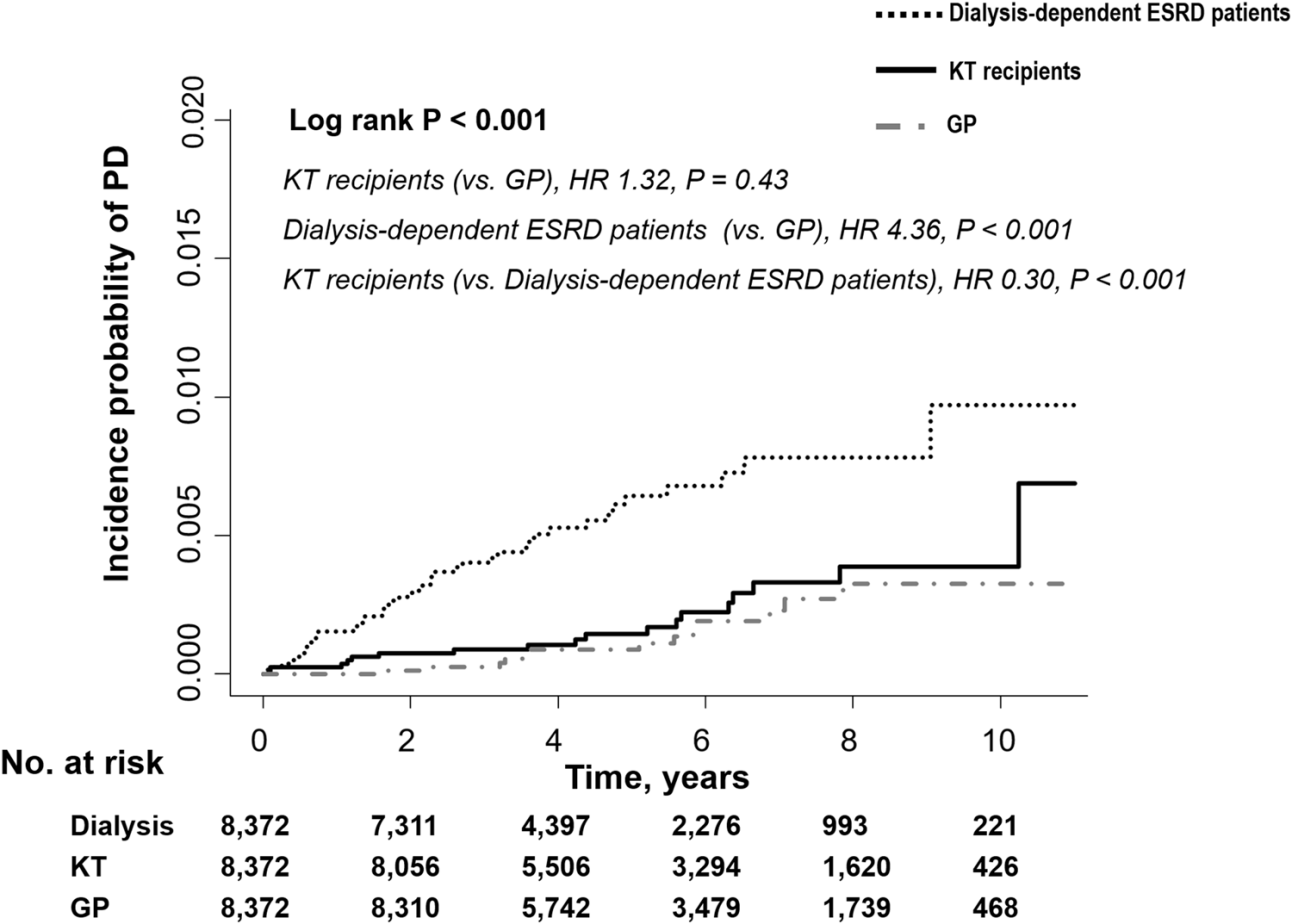
The risk of Parkinson’s disease among the transplant, dialysis-dependent ESRD, and general population groups. ESRD, end-stage renal disease; KT, kidney transplantation; GP, general population; PD, Parkinson’s disease; HR, hazard ratio.

Figure 2 shows the Kaplan–Meier curves of the incidence probability of Parkinson’s disease for up to 10 years in the three groups. Within the observation periods, 19, 53, and 15 KT recipients, dialysis-dependent patients with ESRD, and individuals in the general population developed Parkinson’s disease, respectively. The annual incidence rate of Parkinson’s disease in KT recipients, dialysis-dependent patients with ESRD, and the general population was 0.42, 1.38, and 0.32 per 1000-person-years, respectively. The KT recipients showed a similar and significantly lower risk of incident Parkinson’s disease compared to the general population group (adjusted HR 0.86, 95% confidence interval [CI] 0.35 to 2.13, *P* = 0.75) and dialysis-dependent patients (adjusted HR 0.31, 95% CI, 0.18 to 0.53, *P* < 0.001), respectively. The results remained significant even after adjusting for age, sex, diabetes mellitus, hypertension, dyslipidemia, income, Charlson’s comorbidity index, dialysis vintage period, and use of immunosuppressive treatment before inclusion and nonsteroidal anti-inflammatory drugs (Table 2).

**Table 2:** Risk of Parkinson’s disease.

Group	N	PD events	Follow-up Duration (Person x yr)	Incident Rate (per 1000)	Model^a^		Model^b^		Model^c^	
					HR (95% CI)	*P* value	HR (95% CI)	*P* value	HR (95% CI)	*P* value
**Compared to general population**										
GP	8,372	15	47,475.6	0.32	1 (Ref.)		1 (Ref.)		1 (Ref.)	
KT	8,372	19	45,723.5	0.42	1.32 (0.67,2.59)	0.43	1.34 (0.68,2.63)	0.40	0.86 (0.35,2.13)	0.75
Dialysis	8,372	53	38,356.7	1.38	4.36 (2.45,7.74)	< 0.001	4.61 (2.59,8.19)	< 0.001	2.86 (1.25,6.53)	0.01
**Compared to dialysis-dependent ESRD group**										
Dialysis	8,372	53	38,356.7	1.38	1 (Ref.)		1 (Ref.)		1 (Ref.)	
KT	8,372	19	45,723.5	0.42	0.30 (0.18,0.51)	< 0.001	0.29 (0.17,0.49)	< 0.001	0.31 (0.18,0.53)	< 0.001

Abbreviation: PD, Parkinson’s disease; HR, hazard ratio; CI, confidence interval; KT, kidney transplants; GP, general population.

Model^a^, unadjusted; Model^b^, age, sex; Model^c^, age, sex, diabetes mellitus, hypertension, dyslipidemia, income, Charlson’s comorbidity index, dialysis vintage period, immunosuppressive treatment before inclusion, and use of nonsteroidal anti-inflammatory drugs.

### Predictors of Parkinson’s disease in KT recipients

The KT recipients who developed Parkinson’s disease were older at the time of KT (51.2 vs. 56.0 years old: *P* = 0.003) (see Table 3 and Supplementary Table S1 online). However, incident Parkinson’s disease was not associated with any comorbidities including, diabetes mellitus, hypertension, and dyslipidemia. Moreover, there was no association of incident Parkinson’s disease with income, sex, previous dialysis type and vintage period, and use of immunosuppressive treatment before inclusion (Table 3).

**Table 3:** Risk prediction models for incident Parkinson’s disease in post KT recipients.

Variables	Model^a^		Model^b^	
	HR (95% CI)	*P* value	HR (95% CI)	*P* value
Age, year	1.12 (1.05, 1.19)	< 0.001	1.12 (1.05, 1.20)	< 0.001
Female versus Male	0.38 (0.13, 1.14)	0.09	0.39 (0.13, 1.19)	0.10
**Income (Q)**				
Medical Aid	1 (Ref.)		1 (Ref.)	
No Medical Aid	0.94 (0.27, 3.23)	0.92	0.54 (0.15, 1.95)	0.35
**CCI score**				
1–2	1 (Ref.)	0.96		
3–4	1.70 (0.21, 14.14)			
> = 5	1.77 (0.23, 13.61)			
Diabetes mellitus	0.95 (0.38, 2.33)	0.90		
Hypertension	1.61 (0.22, 12.07)	0.64		
Dyslipidemia	0.65 (0.26, 1.60)	0.25		
**Dialysis vintage period**				
< 5 years	1 (Ref.)		1 (Ref.)	
≥ 5 years	2.38 (0.95, 5.94)	0.06	2.34 (0.93, 5.88)	0.07
**Immunosuppressive treatment before inclusion**				
	0.70 (0.27, 1.79)	0.45	0.68 (0.16, 2.98)	0.61
**Immunosuppressive treatment after inclusion**				
Desensitization	1.83 (0.60, 5.58)	0.29		
**Induction**				
ATG	1 (Ref.)	0.82		
Basiliximab	0.62 (0.14, 2.73)			
**Maintenance CNI**				
Tacrolimus	1 (Ref.)	1.00		
Cyclosporine	1.0 (0.33, 3.05)			

Abbreviation: PD, Parkinson’s disease; HR, hazard ratio; CI, confidence interval; ATG, anti-thymocyte globulin; CNI, calcineurin inhibitor.

Model^a^, unadjusted; Model^b^, age, sex, income, immunosuppressive treatment before inclusion, and dialysis vintage period.

## Discussion

In this nationwide population-based, large-scale, cohort study, we examined the risk of incident Parkinson’s disease in KT recipients compared with that in dialysis patients and the general population. We found that the risk of incident Parkinson’s disease in KT recipients was significantly lower and similar compared with that in dialysis-dependent patients with ESRD and the general population, respectively. Older age at the KT time was an independent risk factor for incident Parkinson’s disease in KT recipients.

We found that the annual incidence rate of Parkinson’s disease in dialysis-dependent patients with ESRD averaged 51-year was 1.38 per 1000-person-year; moreover, they had a significantly higher risk of incident Parkinson’s disease than the KT recipients and the general population. Previous studies have reported an association between chronic uremia and Parkinson’s disease. A 3-year follow-up study in Taiwan based on the Longitudinal Health Insurance Database reported that uremic patients had a 1.81-fold higher risk of incident Parkinson’s disease than nonuremic patients^6^. A 2.56-year follow-up cohort study based on the same database reported an association of dialysis-dependent ESRD with an increased risk of incident Parkinson’s disease (adjusted HR of 1.73, 95% CI 1.39–2.15)^8^. A 5.2-year follow-up cohort study based on the National Health Insurance Service of South Korea reported that an association of a lower estimated glomerular filtration rate and an increased proteinuria severity on dipstick test with a higher occurrence of Parkinson’s disease^7^. Consistent with these previous findings, we found an association of dialysis-dependent ESRD with an increased risk of incident Parkinson’s disease.

In our study, the annual incidence rate of Parkinson’s disease was 0.42 and 0.32 per 1000-person-years in KT recipients and the general population, respectively. This is consistent with the findings of previous studies on the general population aged 50 years^1,17^. Moreover, in our study, the risk of incident Parkinson’s disease in KT recipients was similar to that in the general population. Only one study has assessed the association of incident Parkinson’s disease with organ transplants. In contrast to our results, this previous study, which was based on information obtained from Medicare claims in the United States, reported a lower risk of developing Parkinson’s disease in KT recipients (odds ratio 0.63, 95% CI 0.47–0.84) than that in the general population^12^. They attributed their findings to immunosuppressive agents potentially lowering the risk of Parkinson’s disease given the possible involvement of inflammation in the pathophysiology of Parkinson’s disease^12,18^. However, the analysis of the same database did not yield similar results. In our study, there was no correlation of immunosuppressant usage with the risk of Parkinson’s disease even with thorough analysis and adjustment of confounding factors, including desensitization, induction, and maintenance (data not shown).

KT has been shown to reverse the underlying mechanisms that cause cognitive impairment in CKD, including uremic toxins, hyperparathyroidism, and Klotho deficiency^19^. Initially, as the new transplant begins functioning, ischemia–reperfusion injury causes the up-regulation of pro-inflammatory neurotoxic molecules, uremic toxin removal, restoration of normal calcium-phosphate homeostasis, acute fluid disappearance, and osmotic shift^19^. These positive effects counteract the possible neurotoxic effects of infection^20^ and immunosuppressive drugs, especially corticosteroids and calcineurin inhibitors^21,22^. From this perspective, our findings suggest that dialysis (uremic) patients are at a higher risk of Parkinson’s disease than KT recipients (non-uremic individuals or those with controlled chronic uremia). Consistent with previous reports that KT improves cognitive function^23–27^, our findings suggest that KT may lower the risk of Parkinson’s disease even with long standing kidney disease and/or neurotoxic immunosuppressant usage. Moreover, previous case reports have shown that uremia-associated acute parkinsonism is reversible, as well as the selective vulnerability of the basal ganglia to metabolic derangements, including hypoxia, uremic toxins, and metabolic acidosis^10^. Although the mechanisms underlying the development of Parkinson’s disease in patients with CKD remain unclear, uremia could lead to reversible^10^ and/or permanent destruction of brain structures, including the basal ganglia^9^, and sequelae of movement disorders. Therefore reducing the risk of Parkinson’s disease may also be a reason for recommending KT in older dialysis-dependent patients with ESRD at risk of incident age-related conditions, including neurodegenerative diseases.

This study has several limitations. First, this study involved a short-term follow up of KT recipients, who tended to have a higher risk of incident Parkinson’s disease than that in the general population after 10 years (Fig. 2). Therefore, there is a need for long-term follow-up studies. Second, previous studies have reported that the presymptomatic phase from the onset of neuronal loss to the onset of symptoms was 4.7–5.6 years in Parkinson’s disease^28,29^. The incidence possibility of Parkinson’s disease in dialysis-dependent patients with ESRD seemed to be higher than that in KT recipients at 2 years from the beginning of the study. Neuronal loss may have been ongoing at the start of the present study. Not all dialysis-dependent ESRD patients were on the transplant waitlist, and therefore, potential selection bias remains. To overcome this, history of major diseases including Charlson’s comorbidity index were also analyzed. Third, since the diagnosis of Parkinson’s disease was completely based on information in the claims, there could have been under- or over-estimation of Parkinson’s disease and ambiguity in clinical diagnostic criteria of Parkinson’s disease. Fourth, we analyzed observational data; therefore, it remains unclear why KT recipients are less likely to develop Parkinson’s disease. In our study, the small incidence of Parkinson’s disease impeded evaluation of the association between post-KT renal function and incident Parkinson’s disease. There is a need for future long-term studies on this association to help explain the low incidence of Parkinson’s disease in KT recipients compared with that in dialysis patients. Fifth, since we analyzed only a single ethnic group, our findings cannot be generalized across races. Sixth, due to the limited information obtainable from claim data, we could not adjust for several variables that could affect the occurrence of Parkinson’s disease, including family history of Parkinson’s disease; smoking habits; body mass index; laboratory findings such as serum glucose, creatinine, uric acid, and parathyroid hormone; and dialysis adequacy. Nonetheless, this is the first, large-scale Asian study to compare the incidence of Parkinson’s disease among KT recipients, dialysis-dependent patients with ESRD, and the general population using a nationwide data set in which the insured are covered by a single compulsory insurance with a 100% claim rate. In addition, using a national registry that reports rare intractable disease including Parkinson’s disease leads to yielding more robust entries and the number of patients who are missing is small. Our findings demonstrated that KT was associated with the lower risk of Parkinson’s disease compared with that in dialysis-dependent patients with ESRD. Therefore, KT may be suggested in older dialysis-dependent patients with ESRD at risk of incident Parkinson’s disease^8^.

In conclusion, this study provides evidence that the risk of incident Parkinson’s disease in KT recipients is significantly lower than that in dialysis patients and similar to that in the general population even after adjustment for potential confounding factors. Moreover, we found that older age at the time of KT is an independent risk factor for Parkinson’s disease occurrence in KT recipients.

## Methods

### Data source

The National Health Security System is a mandatory social insurance program in Korea that comprises of the National Health Insurance and Medical Aid^30^. National Health Insurance System, which was launched in 2000 under the supervision of the Ministry of Health and Welfare, is subscribed to by approximately 97% of the Korean population and provides universal health coverage. The remaining 3% of the population is covered by the Medical Aid program. Information on patient demographics, medical service use, disease diagnosis, and life style from both healthcare programs is incorporated into a single National Health Insurance Service database that is accessible for researchers.

In 2006, the South Korean government established a registration program for copayment reduction of up to 10% for patients with rare intractable diseases, including Parkinson’s disease, after a confirmed physician’s diagnosis according to the National Health Insurance diagnostic criteria^7^.

### Study Participants

We analyzed incident KT recipients registered in the Korean National Health Insurance Service database between 2007 and 2015. Moreover, we established two control groups comprised of dialysis-dependent patients with ESRD and individuals in the general population through 1:1 direct matching with the study population. Individuals in the general population without a previous ESRD history were directly matched according to age, sex, and inclusion year. Moreover, dialysis-dependent patients with ESRD without a previous KT history were directly matched according to age, sex, era, hypertension, and diabetes mellitus. During matching, those who failed to find a matched pair were excluded from the study. Subsequently, we excluded matched pairs aged < 40 years, with a history of Parkinson’s disease, or with multi-organ transplantation.

### Study outcomes and follow-up

The primary outcome was incident Parkinson’s disease based on the International Classification of Disease, 10th Revision (ICD-10) codes for Parkinson’s disease (G20) and Parkinson’s disease registration code (V124) during the observation period. The follow-up period lasted from the baseline to the outcome date, death, or December 31, 2017. Additional censoring was conducted for each study group, when KT recipients returned to maintenance dialysis, dialysis patients received KT, or subjects in the general population group received any renal replacement therapy.

### Data collection

We assessed demographic characteristics, including age, sex, and economic status. Economic status was divided into the aid or quartile groups based on the individuals’ insurance fees. We identified baseline comorbidities, including diabetes mellitus, hypertension, and dyslipidemia, based on clinical and pharmacy codes of ICD-10 within 3 years before the inclusion date. We additionally identified microscopic polyangiitis (M31.7), Goodpasture’s syndrome (M31.0), systemic lupus erythematosus (M32), and rapid eye movement sleep behavior disorder (G47.8) based on codes of ICD-10. Moreover, we calculated the Charlson’s comorbidity index^31^. Unique codes given for maintenance dialysis (V001 or O7011-O7020 for hemodialysis, V003 or O7071-7075 for peritoneal dialysis, and V005 or R3280 for transplantation) allow identification of information regarding kidney replacement therapy^32^. We recorded the duration of dialysis before the inclusion date for KT recipients and dialysis-dependent patients with ESRD as categorical variables (< 5 years, ≥ 5 years). We recorded the main dialysis modality as that performed for the longest duration before the inclusion date. Further, if peritoneal dialysis and hemodialysis were performed during similar periods, we recorded the dialysis type as mixed. For the KT recipients, we defined preemptive transplantation as having a previous dialysis duration of < 3 months. To presume the etiologies of ESRD, use of immunosuppressive treatment from 5 year to 6 months before inclusion was obtained (tacrolimus, cyclosporine, mycophenolate mofetil, rituximab, steroids, and cyclophosphamide). Immunosuppressive agent after inclusion (for KT recipients) was recorded as follows: desensitization therapy, including rituximab or plasmapheresis; induction treatment (IL-1 receptor antagonist, anti-thymocyte globulin, or none); and maintenance immunosuppressants except for steroids (tacrolimus, cyclosporine, mycophenolate mofetil, or others). The use of nonsteroidal anti-inflammatory drugs was obtained from the baseline.

### Statistical analysis

We presented baseline characteristics as means and standard deviations for continuous variables and as frequencies (percentages) for categorical variables. Among-group differences in continuous and categorical variables were analyzed using the Mann–Whitney U or Kruskal–Wallis tests and the chi-square or Fisher’s exact test, respectively. We plotted Kaplan–Meier curves to present the cumulative incidence probability of Parkinson’s disease and performed the log-rank test to examine among-group differences. We calculated the incidence of Parkinson’s disease as the number of events per 1000 person-years. We performed Cox proportional hazards analyses to evaluate the risk of incident Parkinson’s disease in KT recipients compared with that in dialysis-dependent patients with ESRD and the general population; moreover, we estimated the HR and 95% CI. A two-sided P value of < 0.05 was considered statistically significant. All analyses and calculations were performed using SAS 9.4 program (SAS Institute, United States).

### Ethical statement

This study was conducted according to the 2008 Declaration of Helsinki and approved by the independent Institutional Review Board of Seoul National University Hospital (IRB number: H1903-098–1018) with no written informed consent because patients records/information was anonymized and de-identified prior to analysis. The need of the Informed Consent was waived by the Institutional Review Board of Seoul National University Hospital.

## Supplementary Information

Supplementary Information.

## Data Availability

All data generated or analyzed during this study are included in this published article.
