# Supplementary material for: Incident Parkinson’s disease in kidney transplantation recipients: a nationwide population-based cohort study in Korea
Source: Sci Rep. 2021 May 18;11:10541. doi: 10.1038/s41598-021-90130-9 (PMC8131700; doi:10.1038/s41598-021-90130-9)
Supplement: Supplementary file 1 — Supplementary Information. [file 41598_2021_90130_MOESM1_ESM.pdf]

Incident Parkinson's Disease in Kidney Transplantation Recipients: A Nationwide Population-Based Cohort Study in Korea

Seon Ha Baek, MD, PhD[1], Sehoon Park MD[2,3], Mi-yeon Yu, MD[4], Ji Eun Kim, MD[5], Sang Hyun Park, MSc[6], Kyungdo Han, PhD[7], Yong Chul Kim, MD, PhD[8], Dong Ki Kim, MD, PhD[8,9], Kwon Wook Joo, MD, PhD[8,9], Yon Su Kim, MD, PhD[2,8,9], Hajeong Lee, MD, PhD[8*]

[1]Department of Internal Medicine, Hallym University Dongtan Sacred Heart Hospital, Gyeonggi-do, Republic of Korea

[2]Department of Biomedical Medicine, Seoul National University Hospital, Seoul, Republic of Korea

[3]Department of Internal Medicine, Armed Forces Capital Hospital, Gyeonggio-do, Republic of Korea

[4]Department of Internal Medicine, Hanyang University Guri Hospital, Gyeonggio-do, Republic of Korea

[5]Department of Internal Medicine, Korea University Guro Hospital, Seoul, Republic of Korea

[6]Department of Medical Statistics, College of Medicine, Catholic University of Korea, Seoul, Republic of Korea

[7]Department of Statistics and Actuarial Science, Soongsil University, Seoul, Republic of Korea

[8]Department of Internal Medicine, Seoul National University Hospital, Seoul, Republic of Korea

[9]Kidney Research Institute, Seoul National University, Seoul, Republic of Korea

Corresponding author contact information

Hajeong Lee, MD, PhD

Department of Internal Medicine, Seoul National University Hospital,

101 Daehak-ro, Jongno-gu, Seoul 03080, Korea.

Tel: 82-2-2072-4905

Fax : 02-762-9662

e-mail: mdhjlee@gmail. com

Supplementary Table S1. Characteristics of kidney transplant recipients by incident post KT Parkinson's disease

Variables	No PD N=8,353	PD N=19	p-value
**Age, mean (SD), y**	51.2 (7.0)	56.0 (7.6)	0.003
**Male (%)**	5013 (60.0)	15 (79.0)	0.09
**Year grading, y (%)**			0.25
2007-2009	1795 (21.5)	7 (36.8)	
2010-2012	2908 (34.8)	6 (31.6)	
2013-2015	3650 (43.7)	6 (31.6)	
**Income (Q) (%)**			0.79
Medical Aid	1146 (13.7)	3 (15.8)	
No Medical Aid	7207 (86.3)	16 (84.2)	
**CCI score (%)**			0.97
0	1 (0.01)	0 (0)	
1-2	698 (8.4)	1 (5.3)	
3-4	2546 (30.5)	6 (31.6)	
>=5	5108 (61.2)	12 (63.2)	
**Diabetes mellitus (%)**	4138 (49.5)	9 (47.4)	0.85
**Hypertension (%)**	7681 (91.9)	18 (94.7)	0.66
**Dyslipidemia (%)**	4961 (59.5)	9 (47.4)	0.29
**Underlying disease**			
Goodpasture's syndrome	3 (0.04)	0	0.93
Microscopic polyangiitis	3 (0.04)	0	0.93
Systemic lupus erythematosus	118 (1.4)	0	0.60
REM sleep behavior disorder	98 (1.2)	0	0.63
**Previous dialysis modality (%)**			0.48
Preemptive	2495 (29.9)	3 (15.8)	
Hemodialysis	3876 (46.4)	12 (63.1)	
Peritoneal dialysis	1422 (17.0)	3 (15.8)	
Mixed	560 (6.7)	1 (5.3)	
**RRT vintage period (%)**			0.14
<5 y	6100 (73.0)	11 (57.9)	
≥5 y	2253 (27.0)	8 (42.1)	
**Immunosuppressive treatment before inclusion (%)**			
	1440 (17.2)	2 (0.53)	0.44
**Immunosuppressive treatment after inclusion (%)**			
**Desensitization**	1354 (16.2)	4 (21.1)	0.57

**Induction**			0.66
No use	348 (4.2)	0 (0)	
ATG	773 (9.2)	2 (10.5)	
Basiliximab	7232 (86.6)	17 (89.5)	
**Maintenance CNI**			0.66
No use	201 (2.4)	0 (0)	
Tacrolimus	6852 (82.0)	15 (79.0)	
Cyclosporine	1300 (15.6)	4 (21.0)	
**Use of NSAID (%)**	1536 (18.4)	5 (26.3)	0.37

Abbreviation: PD, Parkinson's disease; Charlson's comorbidity index; REM, rapid eye movement; ATG, anti-thymocyte globulin; CNI, calcineurin inhibitor; NSAID, nonsteroidal anti-inflammatory drugs
